# Protein disulphide isomerase inhibition as a potential cancer therapeutic strategy

**DOI:** 10.1002/cam4.3836

**Published:** 2021-03-20

**Authors:** Lauren E. Powell, Paul A. Foster

**Affiliations:** ^1^ Institute of Metabolism and Systems Research (IMSR) Medical and Dental School University of Birmingham Birmingham UK; ^2^ Centre for Endocrinology Diabetes and Metabolism Birmingham Health Partners Birmingham UK

**Keywords:** cancer, protein disulphide isomerase, protein disulphide isomerase inhibitors

## Abstract

The protein disulphide isomerase (PDI) gene family is a large, diverse group of enzymes recognised for their roles in disulphide bond formation within the endoplasmic reticulum (ER). PDI therefore plays an important role in ER proteostasis, however, it also shows involvement in ER stress, a characteristic recognised in multiple disease states, including cancer. While the exact mechanisms by which PDI contributes to tumorigenesis are still not fully understood, PDI exhibits clear involvement in the unfolded protein response (UPR) pathway. The UPR acts to alleviate ER stress through the activation of ER chaperones, such as PDI, which act to refold misfolded proteins, promoting cell survival. PDI also acts as an upstream regulator of the UPR pathway, through redox regulation of UPR stress receptors. This demonstrates the pro‐protective roles of PDI and highlights PDI as a potential therapeutic target for cancer treatment. Recent research has explored the use of PDI inhibitors with PACMA 31 in particular, demonstrating promising anti‐cancer effects in ovarian cancer. This review discusses the properties and functions of PDI family members and focuses on their potential as a therapeutic target for cancer treatment.

## Graphical abstract

Protein disulphide isomerases (PDIs) play a vital role in folding proteins into their correct comformation. Inhibition of PDIs can cause ER‐stress leading to cellular apoptosis. In many cancers, PDI expression and activity is increased suggesting they are potential targets for novel therapies. Here we review the latest on PDI in cancer and highlight key PDI inhibitors that may be in the future used as cancer treatments.

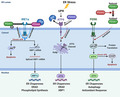

## 1 PDI GENE FAMILY STRUCTURE

The protein disulphide isomerase (PDI) family is a group of multifunctional endoplasmic reticulum (ER) enzymes, recognised to comprise of a total of 21 members.^31^ The number of known human PDI members has increased rapidly over recent years as cDNA sequence data available in the public domain has continued to expand.^3^ Primarily PDIs are recognised to function as a catalyst in the formation, breakage and rearrangement of protein disulphide bonds (S‐S bonds), however, despite the implied isomerase function, not all members have been experimentally proven to demonstrate this activity.^39^ PDI family members are related in that they all possess at least one thioredoxin‐like (TRX‐like) domain.^3^ The TRX‐like domain are categorised into two types; the a‐type (a or aʹ) domain is recognised as catalytically active, while the b‐type (b or bʹ) domain is recognised as catalytically inactive.^36^ Members possessing a‐type domains typically possess a CXXC (Cys‐X‐X‐Cys) motif in an active site, the cysteines are thiol‐reactive and therefore allow for catalytic activity.^36^ CXXC is recognised as the consensus sequence and the most highly conserved catalytic motif is CGHC (Cys‐Gly‐His‐Cys), typically termed the ‘classical’ motif.^1^ While many members contain a combination of both a‐type and b‐type domains in various arrangements, there are some atypical members that only possess one domain type. Members that possess only b‐type domains do not contain cysteines, and hence do not have a catalytic site sequence, they are therefore incapable of meditating S‐S bond formation. These members have been shown to demonstrate a separate function believed to be involved in protein recruitment, with roles as a molecular chaperone.^23^ This demonstrates how PDI members are therefore solely unified on sequence similarity, particularly via the presence of a TRX‐like domain, as opposed to their enzymatic activities. Table 1 demonstrates the diverse nature of the human PDI family, displaying that beyond the presence of a TRX‐like domain, members differ considerably in length, substrate specificity and domain arrangement.^39^

**TABLE 1:** PDI gene family members^41^

Gene Name	Other Aliases	Known Function	Domain Organisation	ER Retention Sequence
PDIA1	P4HB, PDI, PO4DB, ERBA2L	Forms/rearranges disulphide bonds of nascent proteins. Chaperone to inhibit aggregation of misfolded proteins.	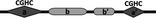	KDEL
PDIA2	PDIp, PDA2	Intracellular estrogen‐binding protein. Chaperone to inhibit aggregation of misfolded proteins.	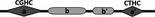	KEEL
PDIA3	ERP57, ERP60, GRP57	Promotes formation of disulphide bonds in glycoprotein substrates. Chaperone to inhibit aggregation of misfolded proteins.	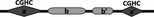	QEDL
PDIA4	ERP70, ERP72	Catalyses protein folding and thiol‐disulphide interchange reactions. Enhances rate of IgG disulphide bonding and antibody assembly when bound to cyclophilin B.	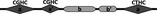	KEEL
PDIA5	PDIR	Catalyses protein folding and thiol‐disulphide interchange reactions. Binding site for ER chaperone calreticulin.	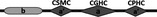	KEEL
PDIA6	P5, ERP5, TXNDC7	Regulates the UPR through binding to and inactivating IRE1 signalling. Chaperone to inhibit aggregation of misfolded proteins.	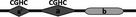	KDEL
PDILT	PDIA7	Chaperone involved in spermatogenesis.	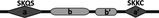	KEEL
ERP27	PDIA8	Specifically binds unfolded proteins and may recruit PDIA3 to unfolded substrates.	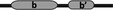	KVEL
ERP29	PDIA9, ERP28	Processes secretory proteins in ER, possibly by folding ER proteins.	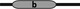	KEEL
ERP44	PDIA10, TXNDC4	Inhibits calcium channel activity of ITPR1. Retains ERO1A and ERO1B in ER and may play role in oxidative folding in ER.	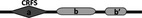	RDEL
TMX1	PDIA11, TXNDC1	Catalyses protein folding and thiol‐disulphide interchange reactions. Cell redox homeostasis.	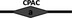	—
TMX2	PDIA12, TXNDC14	Catalyses protein folding and thiol‐disulphide interchange reactions. Cell redox homeostasis.	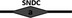	KKDK
TMX3	PDIA13, TXNDC10	Catalyses protein folding and thiol‐disulphide interchange reactions.	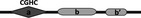	KKKD
TMX4	PDIA14, TXNDC13	Catalyses protein folding and thiol‐disulphide interchange reactions. Cell redox homeostasis.	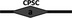	RQR
TXNDC5	PDIA15, ERP46, Endo‐PDI	Catalyses protein folding and thiol‐disulphide interchange reactions. May protect hypoxic cells from apoptosis.	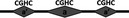	KDEL
TXNDC12	PDIA16, TLP19, AGR1, ERP18, ERP19	Catalyses protein folding and thiol‐disulphide interchange reactions.	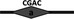	EDEL
AGR2	PDIA17, XAG−2, HAG−2	Catalyses protein folding and thiol‐disulphide interchange reactions. Roles in cell migration, cellular transformation and cell adhesion and is as a p53 inhibitor.	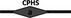	KTEL
AGR3	PDIA18, HAG−3, BCMP11	Catalyses protein folding and thiol‐disulphide interchange reactions. Regulates ciliary beat frequency in multiciliated cells.	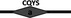	QSEL
DNAJC10	PDIA19, ERDJ5	Co‐chaperone in ERAD. Reduces incorrect disulphide bonds in misfolded proteins, recognised by EDEM1.	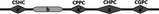	KDEL
CASQ1	PDIB1	Calcium‐binding protein in sarcoplasmic reticulum that acts as an internal calcium store in muscle.	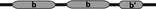	—
CASQ2	PDIB2	Calcium‐binding protein in sarcoplasmic reticulum that acts as an internal calcium store in muscle.	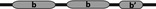	—

**FIGURE 1:**
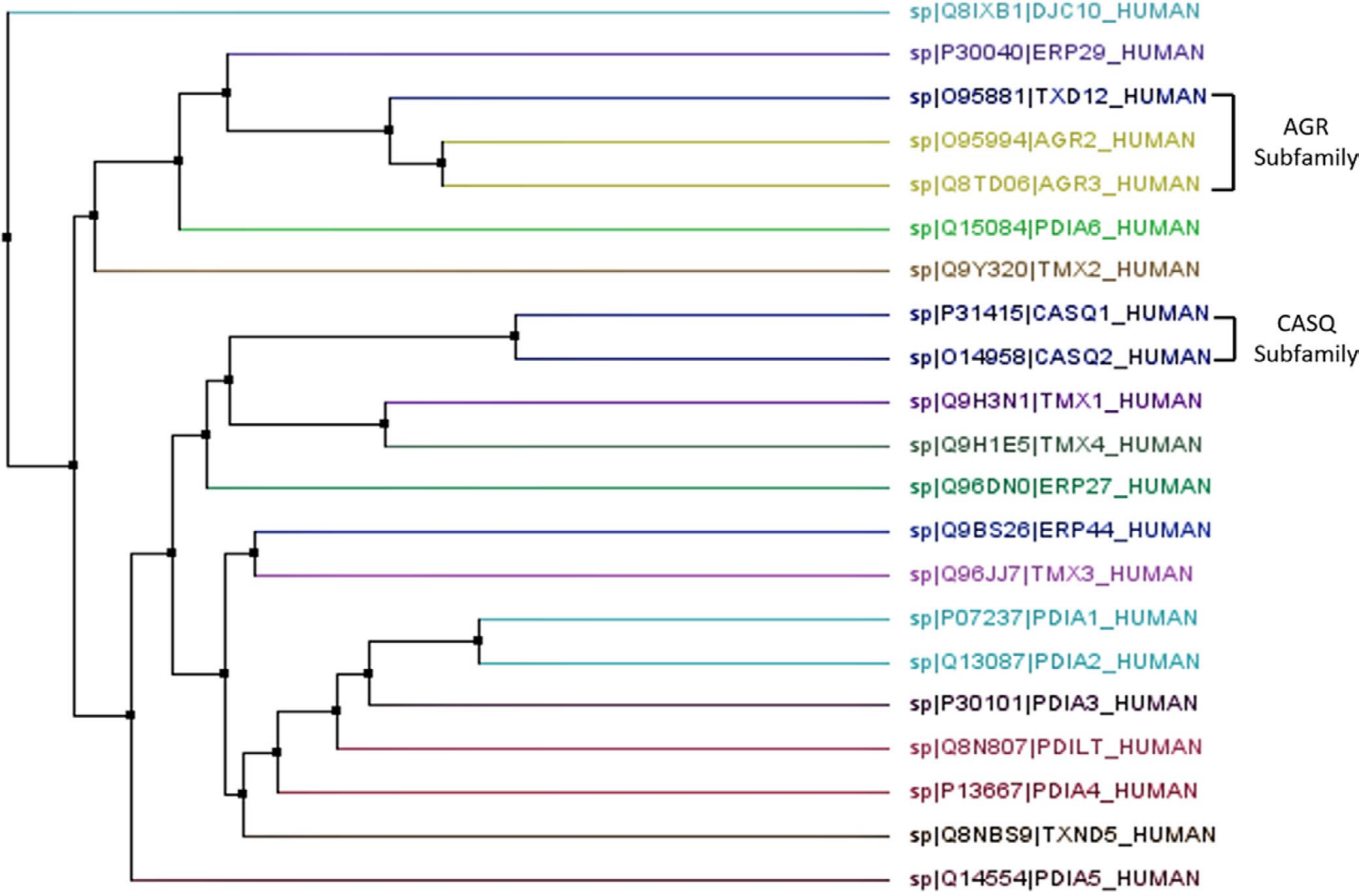
Phylogenetic tree of human PDI gene family. Alignment of protein sequences of human PDI genes was performed using ClustalW software and the phylogenetic tree was constructed by the neighbourhood joining method using Jalview software

As discussed, the presence of a TRX‐like domain is one of the few unifying structural features of PDI family members, suggesting the occurrence of evolutionary divergence among human PDI proteins. Phylogenetic analysis of PDI, shown in Figure 1, demonstrates the existence of PDI subfamilies.^39^ Sequence analysis distinguished both the AGR subfamily and the CASQ subfamily as subsets of genes that appear most evolutionarily related. With the genetic distances represented as estimates of the number of mutations that have accumulated along each branch as they split from the common ancestor, the neighbourhood joining method of phylogenic analysis can only be taken as an approximation. However, the data are supported by the fact that these subfamilies have also been recognised in literature for their structural differences.^39^ The AGR subfamily (AGR2, ARG3 and TXNDC12) is well‐recognised as its members only carry a‐type domains, without the presence of a b‐type domain.^36^ The AGR subfamily members are therefore distinctly recognised for their catalytic role in disulphide bond formation. AGR2 and AGR3 are unique in that while they act to facilitate disulphide bond formation, they lack a C‐terminal cysteine in their catalytic motif and so are recognised to possess non‐canonical CXXS (Cys‐X‐X‐Ser) motifs.^49^ Recent research has demonstrated each cysteine in the consensus CXXC motif to play a unique role; the N‐terminal cysteine acts to form disulphide bonds with the protein substrate; whereas the C‐terminal cysteine plays a role in the release of the substrate.^77^ This suggests that the AGR subfamily may differ from other PDI members in substrate specificity. In contrast, studies have suggested that other PDI members such as PDIA1 and PDIA3 overlap in substrate specificity, with minimal differences.^55^

In contrast, CASQ1 and CASQ2 only possess b‐type domains, and are recognised as their own unique subfamily. The CASQ subfamily act as the main calcium‐binding proteins of the sarcoplasmic reticulum, with their primary role being the regulation of calcium release in muscle, their role in non‐muscle tissues is still unrecognised.^47^ They are also the only PDI members that do not possess an ER retention sequence.^60^ The CASQ subfamily are therefore seen as functionally distinct from other PDI members and could even be recognised to be irrelevant compared to the known functions of other PDI family members. Similarly, ERP27 and ERP29 also only possess b‐type domains, however, they are presumed to play important roles as a molecular chaperone to assist the conformational folding of proteins to assist proteostasis.^2^ ERP29 in particular is recognised as an ER stress‐inducible protein that co‐localises with other ER stress associated chaperones such as BiP.^45^ The existence of these PDI subfamilies therefore reiterates the diverse nature of the PDI gene family and highlights the fact that sequence similarity is the primary determinate factor in the grouping of PDI members, as opposed to their enzymatic properties.

The evolutionary divergence of the PDI gene family is further demonstrated in DNAJC10 which appears dissociated from other PDI members in Figure 1. While it possesses numerous TRX‐like domains, DNAJC10 exhibits no oxidase or isomerase activity and also only possesses approximately one third of the activity that PDIA1 possesses.^66^ Evolutionary divergence is also shown in PDILT, it is recognised as a completely unique member of the PDI family due to the fact that it is specifically expressed in the testis and contains unusual catalytic motifs (SKQS, SKKC) that are not present in any other PDI members.^67^ PDILT has been demonstrated to attribute to S‐S bond formation of the membrane‐bound metalloprotease ADAM3 which acts to aid sperm migration.^64^ Phylogenetic analysis of the human PDI gene family shows that while members are phylogenetically related, a vast amount of evolutionary divergence is apparent. Considering the broad nature of both the domain composition and enzymatic functions, PDI members exhibit quite minimal overall sequence homology.^23^

## 2 PDI FUNCTION

The PDI gene family are highly abundant ER proteins, recognised as folding catalysts.^16^ With the primary function of disulphide bond formation, PDI plays a key role in the correct folding of polypeptide chains in the ER.^23^ The formation of disulphide bonds occurs during oxidative folding in the ER, in which they are formed by the oxidation of thiol groups of cysteines and then isomerised to achieve the correct conformation.^12^ The post‐translational modification that occurs through disulphide bond formation is essential for the stabilisation and maturation of most secretory and membrane proteins in the ER.^53^ PDI therefore plays a critical role in ER proteostasis which aids the maintenance of several cellular functions such as, gluconeogenesis, calcium storage, organelle biogenesis and lipogenesis.^27^ Disruption to ER proteostasis often therefore leads to the progression of multiple disease states through its various influences on cellular stress.^72^

In addition to the role of PDI as a catalyst in disulphide bond formation and rearrangement, PDI proteins also function as molecular chaperones. In this role PDI acts to assist protein folding or refolding via the inhibition of non‐productive folding or by the aggregation of damaged polypeptides or partially folded intermediates.^71^ PDI is believed to inhibit the aggregation of these substrates through interaction with the portions of each substrate that have a tendency to self‐associate; this is achieved by their ability to identify proteins in a non‐naïve conformation.^26^ This role has been suggested to act as an entirely independent function from its role as a catalyst.^75^ PDI still acts to refold misfolded proteins that do not possess disulphide bonds, therefore suggesting the function of PDI as a molecular chaperone and its catalytic activity can be dissociated.^75^

However, PDI can also demonstrate opposing behaviour with aggregation‐prone substrates present at high concentrations, by inducing aggregation of misfolded substrates to in turn precipitate misfolded proteins.^61^ The ability of PDI to facilitate this aggregation as opposed to inhibit it is recognised as anti‐chaperone activity.^52^ ER retention of misfolded proteins has been observed in ER chaperones that possess the KDEL retention sequence which remain in the ER with their unfolded protein cargo.^50^ Misfolded proteins can also become trapped in the ER as large, chaperone‐associated aggregates.^73^ While appropriate folding of these proteins will enable their secretion from the ER, this suggests that the anti‐chaperone activity of PDI contributes to misfolded aggregate formation and accumulation in ER, resulting in significant ER stress.^43^

These observed characteristics of the PDI gene family primarily describe the archetypal PDI (PDIA1), unfortunately less is known about other PDI members. As discussed, although structurally similar, each PDI member does possess a distinct substrate specificity. This has been shown through analysis of disulphide bond formation among major PDI members including PDIA1, PDIA3 and PDIA4, which revealed that each member was specialised for its own unique set of substrates.^32^ For example, PDIA2, which was first identified as a pancreas specific PDI protein, although structurally very similar to PDIA1, is less effective in oxidation, differs in substrate specificity and acts to chaperone denatured substrates.^18^ This demonstrates how PDI members most likely differ in their functions and have divergent function within different tissues.

**FIGURE 2:**
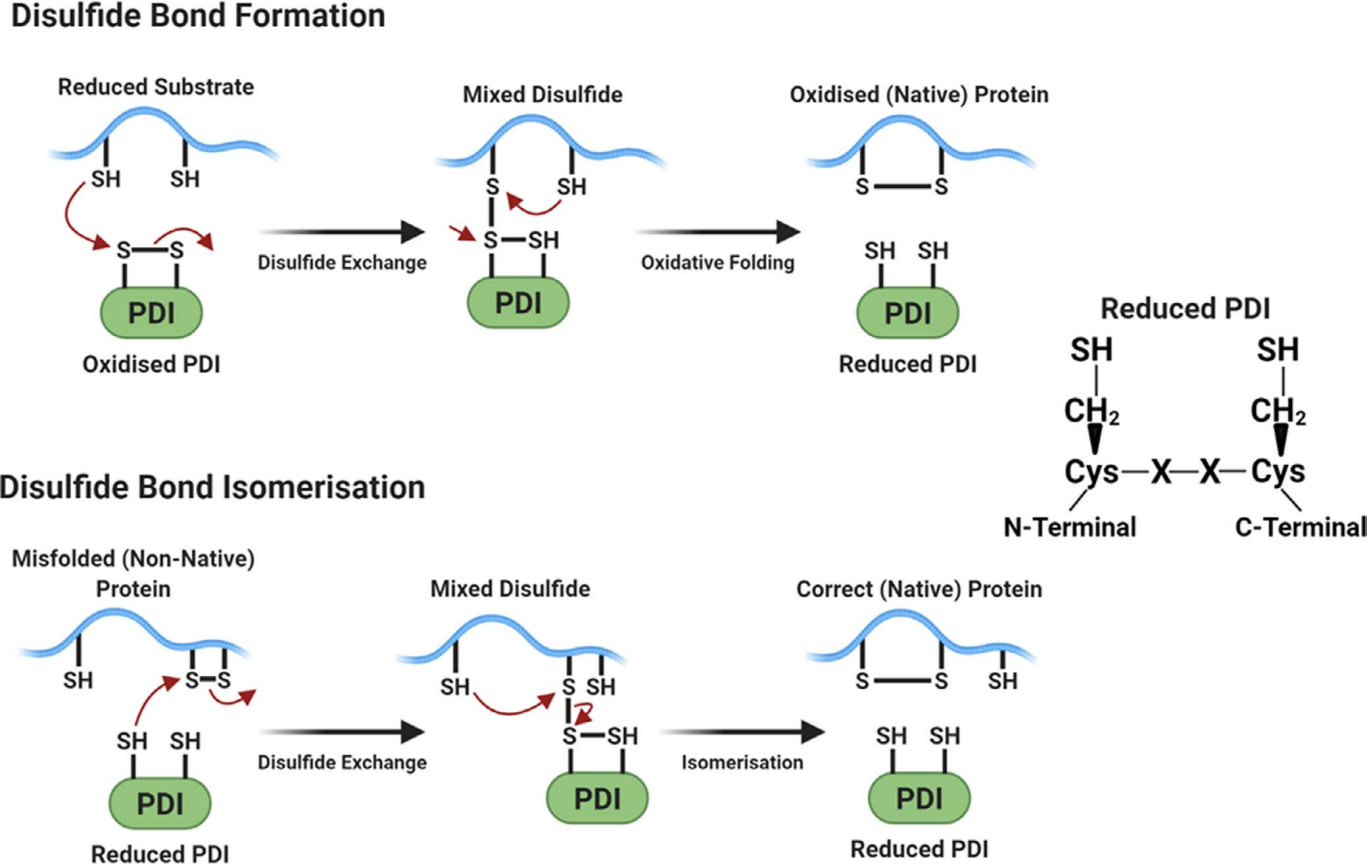
Redox reactions involved with PDI induced disulphide bond formation and isomerisation. (Original Diagram, adapted from Ref. [48]

PDI activity is regulated by redox reactions, with the actions of PDI relying on cycles of reduction and oxidation.^59^ While in a reduced state PDI acts to break non‐native disulphide bonds by process of isomerisation, during an oxidised state PDI acts to correctly introduce and pair cysteines to form native disulphide bonds, and hence both reduced and oxidised pathways lead to the formation of a native protein.^59^ Redox reactions allow PDI to act in such ways through inducing changes in its conformation.^71^ Oxidation acts to alter PDI from a closed conformation to an open conformation, the substrate binding surface therefore becoming more greatly exposed, allowing for a higher activity level and binding affinity.^70^ Reduced PDI remains in a closed, compact conformation which is optimal for binding the substrates that require isomerisation.^71^ Disulphide formation is often error prone during early protein folding resulting in the incorrect pairing of cysteines impeding further folding.^20^ Thus, isomerisation is often required to amend these incorrect cysteines to reform the cysteines to their native arrangement. This described mechanism is portrayed in Figure 2.

## 3 PDI IN ER STRESS

As previously discussed, PDI proteins play a key role in the regulation of proteostasis in the ER, a disruption in PDI activity would therefore result in a disturbance of proteostasis which would in turn trigger an ER stress response. With the accumulation of unfolded and misfolded proteins, ER stress then activates the unfolded protein response (UPR).^39^ Initially, UPR acts to alleviate ER stress via the upregulation of chaperones for protein folding to combat the accumulation of unfolded proteins. The UPR further acts to induce endoplasmic reticulum associated degradation (ERAD) combined with autophagy to remove misfolded proteins.^51^ The UPR therefore does play an important role in combatting ER stress and maintaining cell survival and proteostasis. However, this response is short term, and a prolonged period of ER stress triggers a pro‐apoptotic UPR.^51^ PDI is often found to be upregulated alongside other UPR proteins such as BiP and Grp94, and hence highlights the importance of both UPR and PDI, as a component of UPR, in the regulation of cell survival.^5^ This suggests that prolonged inhibition of PDI would potentially induce apoptosis and thus further highlights PDI as a potential therapeutic target in cancer.

Upon accumulation of unfolded/misfolded proteins, the UPR triggers the activation of three primary ER stress receptors; inositol‐requiring enzyme 1 (IRE1α), activating transcription factor 6 (ATF6) and pancreatic ER kinase (PKR)‐like ER kinase (PERK), shown inFigure 3.^42^ These receptors remain in an inactive state through binding to BiP, an ER chaperone, however, once UPR is triggered by ER stress BiP dissociates from the receptors, resulting in their activation.^56^ The activation of these receptors triggers several pro‐protective pathways to aid cell survival. Activation of PERK induces the transcription of ATF4. ATF4 promotes the expression of ER chaperones such as PDI that refold misfolded proteins.^24^ ATF4 also has further pro‐protective effects through the activation of genes involved in autophagy and an antioxidant response.^8^ Similarly, activation of the IRE1α pathway triggers the splicing of XBP‐1 to induce the accumulation of ER chaperones and ERAD‐associated proteins. IRE1α also regulates phospholipid synthesis which triggers ER membrane expansion to help combat stress.^39^ Upon ATF6 activation, the cleaved ATF6 fragment also induces the expression of XBP‐1, ERAD‐associated proteins and ER chaperones.^58^

**FIGURE 3:**
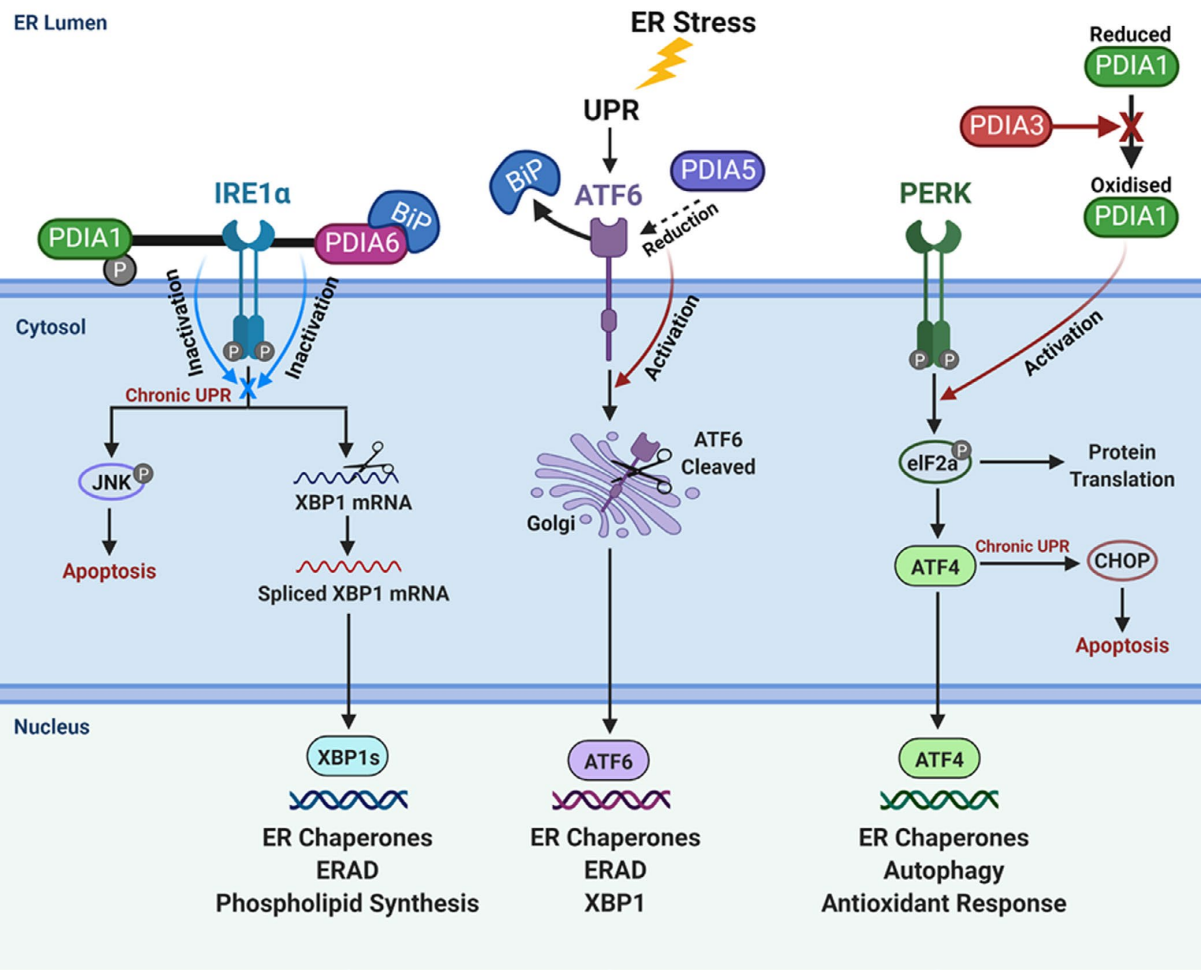
The Unfolded Protein Response Pathway. (Original diagram adapted from Ref. [14, 51, 81]

As discussed, in conditions of prolonged and chronic ER stress, the role of UPR can alter to pro‐apoptotic rather than pro‐survival. Also shown in Figure 3 are the pathways induced following chronic UPR. Prolonged activation of PERK elicits other responses as a consequence of ATF4 activation leading to CHOP upregulation. CHOP plays a key role in ER stress induced apoptosis through its ability to bind to several key UPR proteins, including ATF6 and ATF4.^8^ CHOP also induces ERO1α activation which acts to transfer electrons from PDI to O_2_ to produce hydrogen peroxide, which causes increased oxidative stress.^8^ CHOP can also induce other pro‐apoptotic proteins including p53 upregulated modulator of apoptosis (PUMA) and Bcl2‐interacting mediator of cell death (BIM). Furthermore, CHOP inhibits survival proteins such as BCL‐2.^62^ Interactions with these proteins result in the activation of BAX and BAK associated apoptosis; CHOP is therefore a key player in mediating several apoptotic pathways.^28^ The IRE1α pathway also plays pro‐apoptotic roles under chronic ER stress through the activation of the JNK pathway, which in turn interacts with BAX and BAK; it also induces pro‐inflammatory associated apoptotic pathways.^29^

While PDI plays the role of a pro‐protective downstream ER chaperone, Figure 3 also shows how PDI members also govern UPR activation. The UPR stress receptors; IRE1α, ATF6 and PERK are activated upon directly sensing unfolded protein accumulation. While it is clear that PDI dysfunction leads to protein misfolding, recent studies have also described how PDI acts to mediate redox regulation of the luminal domains of ER stress receptors.^14^ This redox regulation can trigger the activation of the receptors, which in turn triggers the activation of downstream UPR pathways. Research suggests that PDIA5 acts to cleave disulphide bonds in ATF6 leading to its reduction.^30^ The reduction of ATF6, when coupled with BiP dissociation, leads to the ER to Golgi transport of ATF6, inducing the induction of downstream ATF6 target genes.^30^, ^46^ Similarly, PDIA6 acts to exhibit thiol‐disulphide exchange reactions with IRE1α, leading to its reduction.^14^, ^15^ This results in the direct binding of both PDIA6 and BiP to IRE1α which inactivates the IRE1α pathway, therefore acting to prevent exaggerated and chronic UPR signalling.^14^, ^15^ PDIA6 has also been described to interact with PERK, but its interaction is still yet to be fully understood.^14^, ^15^ However, evidence suggests that PDIA3 interacts with PERK through redox regulation, via interaction with PDIA1.^37^ PDIA3 has been described to form a complex with PDIA1 in which it helps to maintain PDIA1 in its reduced state and prevent PERK activation.^3^ The absence of PDIA3 leads to an accumulation of oxidised PDIA1 which triggers PERK activation, suggesting that PDIA3 plays an important role in preventing chronic UPR activation.^37^ Further to this, PDIA1 has been shown to also interact with the IRE1α pathway through alteration in its activity via phosphorylation.^81^ The phosphorylation of Ser357, structurally alters PDIA1 to exhibit an open conformation which helps to prevent protein misfolding and also allows PDIA1 to bind to IRE1α, where it acts to attenuate excessive and chronic UPR signalling.^81^ These recent studies demonstrate various ways in which PDI members are also acting as upstream regulators of the UPR pathway, further demonstrating their pro‐protective properties and potential as therapeutic targets.

## 4 PDI IN CANCER

**FIGURE 4:**
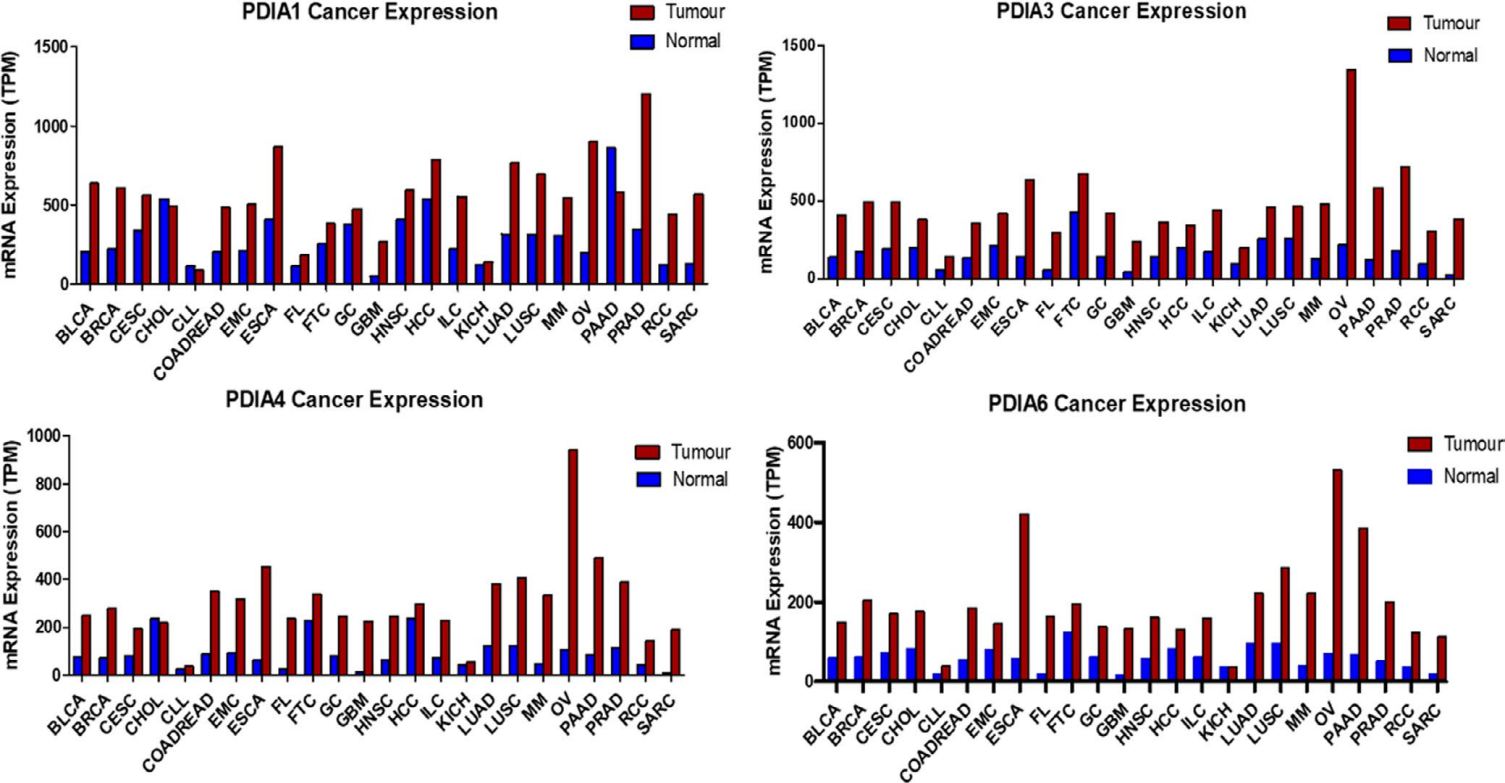
mRNA Expression of PDIA1, PDIA3, PDIA4 and PDIA6 in Tumour vs Normal Tissue across 24 cancer types; see TCGA abbreviations^13^

Although PDI proteins are recognised as one of the most abundant cellular proteins, they are frequently upregulated in a variety of cancer types. Microarray data analysis has demonstrated the upregulation of several typical PDI members including PDIA1, PDIA3, PDIA4 and PDIA6 in multiple cancers such as breast, colorectal, liver, brain and prostate.^10^ This is demonstrated in gene expression data attained from the Gene Expression Atlas datasets.^13^ These data (Figure 4) show a higher level of expression for PDIA1, PDIA3, PDIA4 and PDIA6 in various cancer types compared to normal tissue. The overexpression of PDI is consistent throughout the majority of cancer types and PDI members. This implies that PDI could play an important role in promoting cancer cell survival. However, as these data are based on RNA‐Seq methodology, and that protein phosphorylation plays a major activating role in various PDI pathways which would not be represented here, these data are for guidance only.

The pro‐survival role of PDI has been demonstrated in recent research in lung cancer cells, in which both PDIA4 and PDIA6 were overexpressed and shown to mediate resistance to cisplatin‐induced apoptosis.^65^ Further research has shown that the knockdown of PDIA1 in breast cancer and neuroblastoma cell lines induced cytotoxicity via caspase activation.^25^ This supports the hypothesis that the oncogenic effects of PDI are mediated by their role in the UPR signalling pathway and regulation of apoptosis. An association between PDI and the redox regulation of the UPR sensors has been recognised in recent research and therefore demonstrates the ability of PDI to influence the UPR.^37^ For example, PDIA5 can act to cleave the disulphide bridges of the UPR sensor ATF6 to aid its transport to the nucleus.^30^ ATF6 has been associated with tumour cell resistance to chemotherapy, in particular PDIA5/AFT6 signalling correlates with resistance to imatinib treatment in leukaemia cells, therefore again demonstrating a pro‐survival role of PDI.^30^ Additionally, PDIA6 was demonstrated to directly bind to the luminal domain of the UPR sensor IRE1α through disulphide bond formation, in which it acted to control its activation and aid its return to its inactive state.^14^ PDIA6 therefore acts to attenuate IRE1α and thus alleviates excessive UPR activity.

Whilst PDI is recognised for its roles in UPR signalling and hence cancer cell survival, it also aids the activation of metalloproteases at the cell surface which act to catalyse the shedding of membrane‐associated proteins.^74^ Surface PDI proteins act to catalyse the formation of disulphide bonds through their interaction with client proteins, including integrins, selectins and metalloproteins.^7^ Interestingly, PDI expression is associated with the promotion of metastasis and invasiveness. For example, PDIA1 expression is significantly higher in the metastatic auxiliary lymph node breast tumour than in primary breast tumours.^63^ The catalysation of disulphide bonds in these client proteins by PDI members could be a factor contributing towards the positive correlation between PDI expression and cancer cell metastasis. Furthermore, PDI members may activate membrane proteins such as integrins or proteolytic enzymes such as matrix metallopeptidases (MMPs), both of which mediate cell migration and adhesion and ultimately contribute to metastatic spread.^77^

PDI also contributes towards cancer progression through their involvement in other cancer‐associated signalling pathways, aside from the UPR pathway.^39^ With PDI protein interaction being so vast, each PDI member can be seen as mechanistically distinct. For example, TXNDC5 is recognised to play a role in angiogenesis and is involved in the activation of the Ras‐Raf‐Mek‐Erk (MAPK) pathway. The MAPK pathway is well recognised for its association with various pro‐oncogenic effects; with its activation playing key roles in cell proliferation, apoptosis, differentiation and cell migration, it has become a recent focus of cancer research as a therapeutic target.^57^ This highlights the fact that further research needs to be carried out into the pro‐oncogenic roles of each PDI member, focusing on key associated downstream oncogenic pathways such as UPR, to determine their potential as a therapeutic target for cancer treatment.

## 5 PDI INHIBITORS

There is a clear potential therapeutic role for the use of PDI inhibitors in cancer treatment. Various groups worldwide have identified PDI inhibitors of numerous chemical varieties including both antibiotics and oestrogen polyphenols.^4^ The use of the antibiotic bacitracin as a PDI inhibitor induces apoptosis through the accumulation of ER stress in melanoma cells.^44^ Apoptosis has also been observed in human breast cancer MCF‐7 cells and human neuroblastoma SH‐SY5Y cells as a result of PDI knockdown.^25^ Contradictory to this, PDI knockdown in human cervical cancer HeLa cells did not demonstrate any effects on cell viability.^25^ This suggests that the effects of PDI inhibition on apoptosis may be cell‐type or cancer‐type specific, therefore the development of PDI inhibitors may also be specified to certain cancer types. However, it is unclear from the research which PDI family member is the target of these knockdowns, this reiterates the need for further research into the roles of each specific PDI member to determine their potential as individual therapeutic targets. Bacitracin was additionally shown to inhibit cell migration and invasion in glioblastoma cells, therefore showing the potential of PDI inhibition in combatting metastasis, as well as cancer cell growth and survival.^22^ Despite these promising effects of bacitracin, it is not PDI specific as it inhibits other proteins in the absence of PDI activity.^77^ With bacitracin exhibiting many off‐target effects and although it has been a focus of research, it has not entered clinical trials for PDI‐associated diseases due to its toxicity and poor cell permeability.^21^ Consequently, there is an unmet medical need for the development of function‐specific, small molecule PDI inhibitors for a greater efficacy and more potent treatment.

Propionic acid carbamoyl methyl amides (PACMAs) have been recently recognised as small molecule irreversible PDI inhibitors that have demonstrated cytotoxic effects in a broad range of human cancer cell lines.^79^ Most significantly, a series of PACMA derivatives were demonstrated to result in substantial cytotoxicity in human ovarian cancer cells.^76^ The IC_50 _s for these PACMA derivatives ranged from 0.2 µM to >10 µM, demonstrating a lack of potency across many compounds, with this highlighting the need for the development of compounds with greater efficacy. PACMAs act to irreversibly inhibit PDI by disrupting the PDI CXXC motif through the formation of a covalent bond (C‐S) with the cysteines in the active site.^4^ The disruption of the CXXC motif in PDI appears to be a very proficient method of PDI inhibition. This was demonstrated in PDIA1, which contains two independent active sites, in which the disruption of cysteines in either active site leads to a 50% loss of PDIA1 activity, while disruption at both active sites completely abolishes all PDIA1 activity.^69^ Among these PACMA molecules, PACMA 31 was identified to be an orally active irreversible PDI inhibitor that has demonstrated both oral bioavailability and in vivo activity within a mouse xenograft model of human ovarian cancer.^76^ PACMA 31 has shown anticancer activity both in vitro and in vivo in human ovarian cancer, with no substantial toxicity to normal issue; furthermore, it has also shown effectiveness against chemoresistant cell lines.^76^ This highlights PDI as a druggable target and further promotes research into the use of small molecule PDI inhibitors in cancer treatment.

Table 2 shows several PDI inhibitors and their characteristics of which may have or have previously shown therapeutic potential in cancer treatment. Like PACMA‐31, P1 is a similarly acting, irreversible PDI inhibitor. However, it exhibits a greater potency than PACMA‐31 with an IC50 of 1.7 µM, measured via an in vitro insulin aggregation assay.^19^ P1 also demonstrates inhibition of cell growth across numerous cancer cell lines.^19^ It is again notable, however, that current research has only shown P1 to have the ability to inhibit PDIA1, but this is not selective.^19^ PACMA‐31 on the other hand has shown the ability to bind to and inhibit further PDI members. With a similar mechanism of action, it can be suggested that P1 would also target these members. This reiterates the need to conduct further research to target specific PDI members in order to establish which members are crucial for their role in tumorigenesis. Further observations of the role of PDIA1 in cancer can be made with the use of selective PDIA1 inhibitors; KSC‐34 and RB‐11‐ca. Both of these compounds act to inhibit PDIA1 by inhibiting a and a’ domain sites.^11^ Comparison of these selective PDIA1 inhibitors with E64FC26; a potent pan‐style inhibitor which inhibits; PDIA1, PDIA3, PDIA4, PDIA6 and TXNDC5, would give an indication as to whether inhibition of PDIA1 alone is sufficient for suppressing cancer cell growth.^54^ This then promotes further studies into the selective inhibition of other PDI members.

**TABLE 2:** PDI inhibitor characteristics

PDI inhibitor	Chemical structure	Mode of action	IC50	Known PDI members inhibited	Cell based and pre‐clinical studies	References
Bacitracin	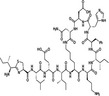	‐ Competitive inhibitor ‐ Binds to free thiols in substrate binding domain ‐ Cell impermeable ‐ Reversible	150–200 µM	PDIA1	Enhances apoptosis in melanoma cells and inhibits migration and invasion of glioblastoma cells.	[17, 40]
PACMA−31	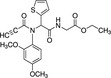	‐ Binds to cysteine residues in active site ‐ Cell permeable ‐ Irreversible	10 µM	PDIA1, PDIA3, PDIA4, PDIA6, TXNDC5.	Inhibits human ovarian cancer cell growth. Inhibits proliferation of OVCAR−8 in culture and in a tumour xenograft model.	[54, 77]
P1	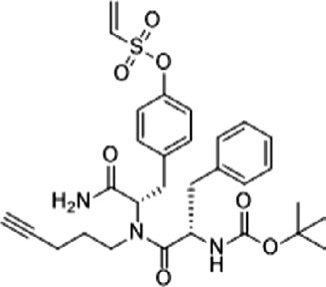	‐ Binds to cysteine residues in active site. ‐ Cell permeable ‐ Irreversible	1.7 µM	PDIA1	Inhibits proliferation of cancer cell lines; MCF−7, Hep‐G2, MDA‐MB−231, UACC−257 , T47D.	[19]
E64FC26	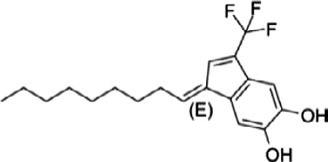	‐ Pan‐style inhibitor ‐ Mechanism unknown ‐ Cell Permeable	1.9 µM	PDIA1, PDIA3, PDIA4, PDIA6, TXNDC5	Induces apoptosis and cytotoxic effects in multiple myeloma cells.	[54]
CCF642	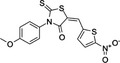	‐ Allosteric inhibitor ‐ Binds to conserved lysine directly adjacent to the active site ‐ Cell permeable ‐ Irreversible	2.9 µM	PDIA1	Induces apoptosis and cytotoxic effects in multiple myeloma cells. Prolongs the lifespan in multiple myeloma mouse model.	[68]
BAP2	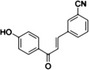	‐ Allosteric inhibitor ‐ Binds to b’ domain ‐ Cell permeable	0.9 µM	PDIA1, PDIA2	Inhibits tumour growth both in vitro and in vivo in glioblastoma.	[78, 80]
35G8	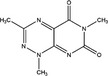	‐ Mechanism Unknown	0.17 µM	Represses PDI target genes such as TXNIP and EGR1.	Induces cell death via autophagy and ferroptosis in glioblastoma cells.	[38]
LOC14	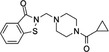	‐ Allosteric inhibitor ‐ Binds adjacent to active site, forces protein to maintain oxidized conformation ‐ Cell permeable ‐ Reversible	5 µM	PDIA3	Antiapoptotic, neuroprotective function on nerve cells in a model of Huntington disease.	[9, 34]
16F16	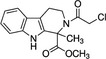	‐ Binds to cysteine residues in active site ‐ Cell permeable ‐ Irreversible	∼70 μM	PDIA1, PDIA3	Prevent apoptosis induced by mutant huntingtin protein and neuroprotective in rat neurons.	[19]
KSC−34	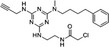	‐ Selective PDIA1 inhibitor ‐ Inhibits C53 in a domain active site. ‐ Cell permeable	N/A	PDIA1	30‐fold selectivity for the a‐site over the a’ site and shows time‐dependent inhibition of PDIA1 reductase activity in vitro.	[11]
RB−11‐ca	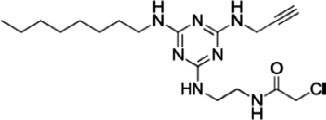	‐ Selective PDIA1 inhibitor ‐ Inhibits C53 in a domain active site. ‐ Cell permeable ‐ Irreversible	30–50 µM	PDIA1	Inhibits proliferation of HeLa cells.	[11]
ML359	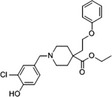	‐ Likely binds to b’ domain ‐ Cell permeable ‐ Reversible	0.25 µM	PDIA1	Inhibits platelet aggregation. Not cytotoxic in human cell lines.	[6, 35]
Juniferdin	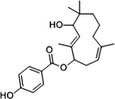	‐ Mechanism unknown. ‐ Cell permeable ‐ Reversible	0.16 µM	PDIA1	Inhibits reduction of HIV−1 gp120 and reduces influenza virus replication. Cytotoxic in several cell lines.	[35]
Quercetin−3‐rutinoside	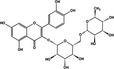	‐ Binds to b’ domain ‐ Cell impermeable ‐ Reversible	6 µM	PDIA1	Inhibits platelet aggregation and blocks thrombus formation in vivo. Not cytotoxic.	[33]

Table 2 demonstrates how different PDI inhibitors vary in their mode of action. While KSC‐34 and RB‐11‐ca specifically inhibit a‐type domains of PDIA1, BAP2 specifically inhibits b‐type domains of PDIA1.^78^ BAP2 has demonstrated apoptotic effects in glioblastoma cells, therefore comparison with KSC‐34 and RB‐11‐ca could give insight into the roles of catalytic a‐type sites and non‐catalytic b‐type sites in driving cancer cell growth. Research has also shown 35G8 to have anti‐cancer effects in glioblastoma through the induction of cell death via autophagy and ferroptosis rather than apoptosis.^38^ Although the mechanism of action of 35G8 is unknown, it appears to lack drug‐like properties as rather than specifically targeting PDI, it acts to repress PDI target genes such as TXNIP and EGR1.^38^ 35G8 is also recognised as a redox cycling molecule.^78^ This suggests a potential use of 35G8 as an adjuvant in combination with other PDI inhibitors such as BAP2 as a potential therapeutic strategy. The vast range of PDI inhibitors promotes further research into the discovery of the most effective mode of action of PDI inhibition and the most important PDI members to target in order to develop novel drugs and improved therapeutic strategies.

## 6 CONCLUSIONS

The PDI family consists of large, complex group of proteins, with varying roles and functions. While PDI is recognised for its role in ER proteostasis through the catalysation of disulphide bonds, it has also been recognised to exhibit effects in several disease states, in particular cancer. It is clear that PDI involvement in ER stress impacts both cancer cell survival and apoptosis due to the complex nature of the UPR pathway; it can have protective and detrimental downstream effects. The involvement of PDI in each of these downstream pathways requires further research to isolate further potential gene targets involved in cancer cell survival. PDI proteins are a clear therapeutic target in cancer treatment, with several PDI inhibitors demonstrating anticancer effects. Thus, there is substantial potential for the development of further small molecule PDI inhibitors for therapeutic use in a variety of cancer types. Further research is also needed in order to determine the key PDI family members involved in the promotion of tumorigenesis due to their vast and diverse nature. The precise targeting of key PDI proteins within a specific cancer type has the potential to provide a more effective, personalised treatment strategy.

## CONFLICT OF INTEREST

The authors do not have any conflict of interest.

## AUTHOR CONTRIBUTION

LEP and PA performed literature searches and wrote the manuscript. LEP analysed data for Figure 4.

## ETHICAL APPROVAL

Ethical approval was not required for this work.

## Data Availability

The data that support the findings of this study are available in National Cancer Institute's Cancer Genome Atlas Program at https://www.cancer.gov/about‐nci/organization/ccg/research/structural‐genomics/tcga.
